# Structural, Functional and Phylogenetic Analysis of Sperm Lysozyme-Like Proteins

**DOI:** 10.1371/journal.pone.0166321

**Published:** 2016-11-10

**Authors:** Shalini Kalra, Mangottil Ayyappan Pradeep, Ashok K. Mohanty, Jai K. Kaushik

**Affiliations:** BTIS Sub-DIC, Animal Biotechnology Centre, National Dairy Research Institute, Karnal, 132001, India; NCI at Frederick, UNITED STATES

## Abstract

Sperm lysozyme-like proteins belonging to c-type lysozyme family evolved in multiple forms. Lysozyme-like proteins, *viz*., LYZL2, LYZL3 or SLLP1, LYZL4, LYZL5 and LYZL6 are expressed in the testis of mammals. Not all members of LYZL family have been uniformly and unambiguously identified in the genome and proteome of mammals. Some studies suggested a role of SLLP1 and LYZL4 in fertilization; however, the function of other LYZL proteins is unknown. We identified all known forms of LYZL proteins in buffalo sperm by LC-MS/MS. Cloning and sequence analysis of the *Lyzl* cDNA showed 38–50% identity at amino acid level among the buffalo LYZL paralogs, complete conservation of eight cysteines and other signature sequences of c-type lysozyme family. Catalytic residues in SLLP1, LYZL4 and LYZL5 have undergone replacement. The substrate binding residues showed significant variation in LYZL proteins. Residues at sites 62, 101, 114 in LYZL4; 101 in SLLP1; 37, 62, and 101 in LYZL6 were more variable among diverse species. Sites 63 and 108 occupied by tryptophan were least tolerant to variation. Site 37 also showed lower tolerance to substitution in SLLP1, LYZL4 and LYZL5, but more variable in non-testicular lysozymes. Models of LYZL proteins were created by homology modeling and the substrate binding pockets were analyzed in term of binding energies and contacting residues of LYZL proteins with tri-N-acetylglucosamine (NAG)_3_ in the A-B-C and B-C-D binding mode. Except LYZL6, LYZL proteins did not show significant difference in binding energies in comparison to hen egg white lysozyme in the A-B-C mode. (NAG)_3_ binding energy in the B-C-D mode was higher by 1.3–2.2 kcal/mol than in A-B-C mode. Structural analysis indicated that (NAG)_3_ was involved in making more extensive interactions including hydrogen bonding with LYZL proteins in B-C-D mode than in A-B-C mode. Despite large sequence divergence among themselves and with respect to c-type lysozymes, substrate binding residues as well as hydrogen bonding network between (NAG)_3_ and proteins were mostly conserved. LYZL5 in buffalo and other mammalian species contained additional 10–12 amino acid sequence at c-terminal that matched with ankyrin repeat domain-containing protein 27. Phylogenetic analysis indicated LYZL2 to be most ancient among all the LYZL proteins and that the evolution of LYZL proteins occurred through several gene duplications preceding the speciation of mammals from other vertebrates as distant as reptiles and amphibians.

## Introduction

Lysozymes are hydrolytic enzymes, which cleave the β-(1,4)-glycosidic bond between N-acetylmuramic acid and N-acetylglucosamine (NAG) residues of peptidoglycan of bacterial cell wall. It can also cleave β-(1,4) glycosidic bond of polymer of NAGs in chitin which is found in cell wall of fungi, exoskeletons of crustaceans, arachnids and insects [1,2]. On the basis of biological origin, lysozymes are divided into several families: chicken (c-type), goose (g-type), invertebrate (i-type), bacteriophage and plant lysozymes. Among these, c-type lysozymes are the most common and widely distributed among insects, fishes, reptiles, birds and mammals. Lysozyme is secreted in many body fluids such as saliva, blood, tears and milk in mammals. It is present at very high concentration in the eggs of many bird species [3]. Lysozyme expression has also been observed in many other tissues, *viz*., stomach, kidney, lacrimal gland, parotid gland, sublingual gland, lung, spleen, small intestine, lymph node, placenta and leukocytes [4].

Lysozyme has evolved in different tissues to perform specific functions, e.g., it has evolved as a digestive enzyme in ruminants [5,6], leaf eating monkeys [7] and hoatzin bird [8]. The stomach lysozyme has evolved to resist the acidic environment and pepsin in stomach. Ruminant artiodactyls like cattle and sheep have approximately ten lysozyme genes, whereas non-ruminant artiodactyls like pig have only single gene [9–11]. The c-type lysozyme family also includes α-lactalbumin and calcium-binding lysozyme. α-lactalbumin shares 40% sequence similarity with lysozyme but lacks bacteriolytic activity [12]. It is restricted to mammals and expressed specifically in lactating mammary glands to help in the synthesis of lactose [13]. Bacteriolytic calcium-binding lysozymes are found in the milk of few mammalian species (e.g. in dog, cat, seal, horse and echidna) and in the eggs (e.g. pigeon) and stomach of some leaf eating bird species like hoatzin [14]. A pseudogene *LYSC1* with calcium-binding property has also been found in some mammalian species [15]. It has been thought that calcium-binding lysozymes evolved from lysozyme before the divergence of birds and mammals; however, α-lactalbumin evolved only in mammalian clade.

Recently testis specific lysozyme-like genes (*Lyzl*) belonging to the c-type lysozyme family have also been discovered. Five forms of lysozyme-like genes *Lyzl2*, *Lyzl3/Spaca3*, *Lyzl4*, *Lyzl5/Spaca5* and *Lyzl6* have been identified in the testis of human and other mammalian species [16–19]. The lysozyme-like proteins (LYZL) along with commonly known lysozyme have been identified from sperm proteome of mouse and human [20,21]. LYZL4 protein has also been observed on the surface of human embryonic stem cells [22]. LYZL5/SPACA5 protein has been associated with Type-II diabetes as well [23]. Lysozyme, LYZL4, LYZL3/SPACA3 and LYZL5/SPACA5 have also been identified in the human sperm tail proteome [24]. LYZL3/SPACA3, LYZL4 and LYZL5/SPACA5 have been suggested to serve as biomarkers for male fertility [25–29]. LYZL3/SPACA3 protein is also known as sperm lysozyme-like protein 1 (SLLP1).

Lysozyme-like genes possess genomic organization and conserved signature sequences that are common among lysozyme family members [15,17]. In several LYZL proteins, the catalytic residues common among active c-type lysozymes are not conserved [16,18,30]. The role of multiple *Lyzl* genes in the male reproductive system is still obscure. The first LYZL protein SLLP1 encoded by *Spaca3* gene was reported to have a role in sperm-egg binding in human [16] and mouse [31]. Recently, presence of SLLP1 was observed in bovine oocytes and implicated in female fertility as well [32]. Mouse LYZL4 was also reported to be involved in male fertility [30]. LYZL1/LYZL2 and LYZL4 protein expression was found downregulated in asthenozoospermic condition in human [33]. Expression of several *Lyzl* genes has been observed in tissues like brain, lung, heart, spleen, kidney, ovary and uterus [17–19]; however their tissue-specific role is almost unknown. Functional characterization of LYZL proteins has been restricted mainly to human and mouse in case of SLLP1 [16,31] and mouse in case of LYZL4 [30]. The functions of LYZL2, LYZL5 and LYZL6 are yet to be uncovered.

The evolution of numerous *Lyzl* genes and their functional role in mammals is still not well understood. Not all *Lyzl* genes are invariably distributed in all mammals and other vertebrates. In the present investigation, we identified all the known forms of lysozyme-like proteins in buffalo sperm lysate. The identified *Lyzl* genes were cloned, sequenced and comparative sequence analysis carried out to understand the effect of amino acid replacements on LYZL function. Further, structural models were built for LYZL proteins complexed with lysozyme inhibitor tri-N-acetylglucosamine (NAG)_3_ to understand the structural and functional properties of the lysozyme-like proteins.

## Materials and Methods

### Isolation of total sperm proteins

Fresh semen sample of riverine buffalo (*Bubalus bubalis* Murrah breed) was obtained in sterile tubes from the semen collection service of the Artificial Breeding Research Centre, National Dairy Research Institute. The bull semen is regularly collected at the centre for breeding program or for discarding to maintain good semen quality in the bulls and hence no ethical clearance was required for use of semen sample. The semen was centrifuged at 9500×g for 10 min at 4°C. The supernatant was discarded and the pellet with sperm was washed with PBS buffer at pH 7.4 several times and mixed with radio immunoprecipitation assay lysis buffer (Cat. No. R0278, Sigma Chem. Co., USA) and incubated on ice for 45 min [34]. Thereafter, the mixture was centrifuged at 16000×g for 30 min at 4°C and the supernatant fraction was run on 12% SDS-PAGE and the gel was stained with 0.1% Coomassie brilliant blue G-250 in 30% methanol and 10% acetic acid.

### Mass spectrometry analysis

The gel slices corresponding to sperm proteins of 12–18 kDa molecular weight were excised from acrylamide gel. Trypsin digestion of the extracted proteins was performed as described for in-gel digestion [35]. The peptide mixture was fractionated on a liquid chromatography (LC) system equipped with nano-trap column (C18, 5 μm, 200 Å, 0.1 x 150 mm) and a reverse phase nano-column (C18, 3 μm, 200 Å, 0.1 x 150 mm) in series. The mobile phase included 0.1% formic acid in water and 99.9% acetonitrile with 0.1% formic acid. A linear gradient of acetonitrile was set from 5% to 45% in 30 min at a flow rate of 800 nl/min. The peptides were submitted to the inline nanospray equipped ESI-tandem mass spectroscopy (MS/MS) maXis HD ESI-QUAD-TOF (Bruker Daltonics, Germany). The mass spectrometer was configured for data-dependent acquisition mode for automatic selection of precursor ions for further analysis by MS in the m/z range of 50–2200. The generated MS spectra were used for searching against NCBI database by using Mascot software (Matrix Science). The peptide mass tolerance and MS/MS tolerance were set to 50 ppm and 0.05 dalton, respectively in the Mascot search.

### Cloning of buffalo *Lyzl* genes

The primer pairs for the amplification of lysozyme-like genes were designed from cattle gene sequences with NCBI accession numbers BC126794 (*Lyzl2*), BC149686 (*Lyzl3*/*Spaca3*), BC111339 (*Lyzl4*), BC108157 (*Lyzl5*/*Spaca5*) and BC114039 (*Lyzl6*). Primers were designed from the regions outside the open reading frames (ORF) by using Primer3 software.

Buffalo testicular tissue was collected from the adult males slaughtered for the meat purpose at the Delhi slaughter house, Ghazipur, New Delhi. Animals were not slaughtered specifically for tissue collection and hence ethical clearance was not required to collect the samples. Total RNA was extracted from the testicular tissue by using Trizol reagent (Invitrogen, USA) as per manufacturer’s instructions. Phusion RT-PCR kit (Finnzymes, Finland) was used for the synthesis of first strand cDNA. Full strand cDNA was amplified by using gene specific primers and the PCR product was ligated with pJET1.2/blunt vector (Fermentas, USA) followed by transformation into chemically competent TOP10 cells (Invitrogen, USA). At least two positive clones confirmed by gene-specific PCR were used for sequencing. The recombinant DNA related procedures were approved by the local institute biosafety committee.

### Protein sequence analysis

The ORF in various *Lyzl* genes were determined by using the NCBI ORF finder tool (http://www.ncbi.nlm.nih.gov/projects/gorf/) followed by prediction of amino acid sequence using ExPASy translate tool (http://www.expasy.org). The signal peptide was predicted by using online SignalP 4.0 server (http://www.cbs.dtu.dk/services/SignalP/) based on neural network trained on eukaryotes [36]. The amino acids composition, molecular weight and isoelectric point (p*I*) of matured lysozyme-like proteins were predicted by using ExPASy ProtParam tool (http://expasy.org/protparam/). Signature sequence pattern of a lysozyme-like protein was identified with PROSITE database at ExPASy [37].

The multiple sequence alignment of buffalo LYZL proteins was performed by using the ClustalW2 program (www.ebi.ac.uk/Tools/msa/clustalw2) and pairwise sequence identities were determined between the orthologs and paralogs. The protein sequences of LYZL proteins of several species were retrieved from UniProtKB database (ExPASy) for sequence analysis. Non-testicular lysozymes included hen egg white lysozyme (HEWL), turkey egg white lysozyme (TEWL), hotzain bird, rainbow trout lysozyme (RBTL), Japanese quail, solea fish lysozyme and lysozymes from milk, stomach, mammary gland and also from other tissues of several species. Testicular lysozyme-like proteins included LYZL2, SLLP1, LYZL4, LYZL5 and LYZL6 from human, chimpanzee, gorilla, gibbon, baboon, monkey, orangutan, garnett’s greater bushbaby, mouse, rat, mole rat, buffalo, cattle, sheep, goat, cat, dog, ground squirrel, rabbit, guinea pig, giant panda, mustela furo, african elephant, horse, camel, little brown bat, black flying fox, chinese hamster, american pika, armadillo, and european shrew (S1 Table). Protein sequences were analyzed for the identification of residues involved in catalysis and substrate binding. The substrate binding residues were analyzed among non-testicular lysozymes and testicular lysozyme-like proteins from several species.

For phylogenetic analysis, sequences were aligned with muscle program by using default parameters [38]. The phylogenetic tree of lysozyme-like proteins was generated with MEGA6 software [39] by using maximum likelihood method (ML) based on LG model [40] and by using a bootstrap value of 1000. Initial tree was obtained by heuristic search using Neighbor-Joining method as implemented in MEGA6 program. Five discrete gamma distribution categories were taken into account for evolutionary rates among sites.

### Structural modeling of LYZL-(NAG)_3_ complexes and estimation of binding energies

Homology models of LYZL proteins complexed with (NAG)_3_ were built with YASARA program (version 16.4.8) [41] by using the protocol similar to that used earlier [42]. (NAG)_3_ has been reported to bind in A-B-C or B-C-D subsites of c-type lysozymes, therefore, LYZL complex models with (NAG)_3_ were created in both binding modes. The following template structures, HEWL (PDB IDs: 1LZB [43], 1HEW [44], 3A3Q [45]), TEWL (PDB ID: 1JEF) [46], human lysozyme (PDB ID: 1BB5) (unpublished) containing (NAG)_3_ in the A-B-C binding mode were used for LYZL-(NAG)_3_ in the A-B-C mode. PDB ID 1BB5 contained two molecules and hence both were used in making the models after separating them. For B-C-D mode, complex structures with well-defined positions of (NAG)_3_ were not available for HEWL, TEWL or human lysozyme. Superimposition of RBTL-(NAG)_4_ complex structure in A-B-C-D binding mode (PDB ID: 1LMQ) [47] with RBTL-(NAG)_3_ complex in the B-C-D mode (PDB ID: 1LMP) [47] showed NAG units in the B-C subsites to be completely overlapping [48] with minor change in NAG conformation at subsite D [47]. Similar observation has been made in HEWL complex, where B-C subunits of (NAG)_4_ completely superimposed with that of (NAG)_3_ in the partially occupied A-B-C and B-C-D binding modes [43], and with minor flexibility in position of NAG at subsite A. Therefore, we used HEWL (PDB ID: 1LZC) [43], (PDB ID: 1LSZ) [49], (PDB ID: 3WVY) [50], human lysozyme (PDB ID: 1LZR) [51] and RBTL (PDB ID: 1LMQ) [47] with (NAG)_4_ in the A-B-C-D subsites and deleted NAG in subsite A to produce lysozyme-(NAG)_3_ in B-C-D subsites. The RBTL complex structure with B-C-D binding mode (PDB ID: 1LMP) was also used as a template without any change.

The sequences of LYZL proteins along with the above complex templates were manually provided and alignment profiles were built by running PSI-BLAST [52]. Single model was created if alignment was certain, otherwise alternative models were built when alignment was ambiguous with a given template sequence. The missing loops were built and the side-chain rotamers optimized by taking into account electrostatic interactions, knowledge-based packing interactions and the solvation effect [53]. Initially, energy minimization was carried out with combined steepest descent and simulated annealing by fixing the backbone atoms of the aligned model. Thereafter, minimization with full unrestrained all-atom simulated annealing was carried out. The protocol used implicit solvent model for the optimization of side-chains and loops and explicit solvent shell model for the simulated annealing to fine tune the models. The overall quality Z-score, which is defined as the weighted averages of the individual Z-scores, was calculated by using the equation, Z-score = 0.1456 × Dihedrals + 0.3906 × Packing1D + 0.4656 × Packing3D [53]. The models with lower quality Z-score were discarded. The detailed stereochemical quality checks of the models were also carried out by using Procheck program.

The complex structures of LYZL with (NAG)_3_ were superimposed and aligned by using MUSTANG program implemented in the YASARA program [54] and the RMSD of the models was calculated with respect to template structure. The selected models based on structural alignment and quality Z-score were further refined by all-atom MD using Yasara2 force field [53] with explicit solvent model. The periodic cell was filled with a water density of 0.997 and p*K*_a_ of titrable side chains was predicted [55] and assigned to respective groups before running MD. The MD was run at 298 K by controlling temperature with Berenden-type thermostat as implemented in the YARASA program where temperature of the solute and water were controlled independently to achieve uniform temperature in the cell. The trajectories were calculated with a time step of 2 fs and snapshots were saved at every 25 ps. The MD for refinement of all the initial homology models was run for 250 ps. For calculating binding energies of (NAG)_3_ with LYZL proteins, all the 10 snapshots obtained during refinement for a given template were used to avoid bias toward specific conformation. Finally, binding energies of all the conformations generated from various templates were averaged out to avoid conformational and template biasness. Before calculating the binding energies of (NAG)_3_ with LYZL proteins, hydrogen bonding network was optimized for all the LYZL-(NAG)_3_ complex structures by using the Yasara method [56]. Binding energy and contacting residues of templates and LYZL-(NAG)_3_ complexes were calculated by using molecular docking program VinaDock [57] with default parameters. The MD and docking protocol were set up in YASARA program [54].

## Results

### Identification of LYZL proteins in buffalo partial sperm proteome

LYZL proteins are constituted of 126–130 amino acid residues with molecular mass of 14–15 kDa. To identify lysozyme-like proteins in buffalo sperm, we analyzed sperm proteome subset corresponding to 12–18 kDa molecular mass by using LC-MS/MS. All LYZL proteins namely LYZL2, SLLP1, LYZL4, LYZL5 and LYZL6 could be identified in Mascot search.

### Cloning and sequence analysis of *Lyzl* genes

The PCR amplified products of 519 bp (*Lyzl2*), 557 bp (*Lyzl3*/*Spaca3*), 532 bp (*Lyzl4*), 532 bp (*Lyzl5/Spaca5*) and 547 bp (*Lyzl6*) cloned into blunt end PCR cloning vector were sequenced. The sequences were deposited in NCBI nucleotide database with accession numbers JN130361 (*Lyzl2*), JN130363 (*Lyzl3*/*Spaca3*), HQ285241 (*Lyzl4*), JN130364 (*Lyzl5*/*Spaca5*) and JN130362 (*Lyzl6*). The open reading frame sizes of these genes were determined as follow, 447 bp (*Lyzl2*), 492 bp (*Lyzl3*/*Spaca3*), 438 bp (*Lyzl4*), 471 bp (*Lyzl5*/*Spaca5*) and 447 bp (*Lyzl6*). Precursors of LYZL2 and LYZL6 proteins consisted of N-terminal signal peptide of 19 amino acids and mature protein of 129 amino acids. SLLP1, which is a product of *Lyzl3*/*Spaca3* gene, possessed a signal peptide of 35 amino acids and mature protein of 128 amino acids. LYZL4 protein consisted of 19 residues in signal peptide and 126 residues in mature protein. LYZL5 consisted of a signal peptide of 18 amino acids and mature protein of 138 amino acids. SLLP1, LYZL5 and LYZL6 were observed acidic in nature with isoelectric points (p*I*) of 5.22, 5.55 and 5.15, respectively, whereas LYZL2 and LYZL4 were basic with p*I* of 8.13 and 8.46, respectively.

Pairwise sequence comparison showed that various buffalo LYZL proteins shared only 38–50% sequence identity with each other and other non-testicular c-type lysozymes like HEWL, human lysozyme, buffalo milk lysozyme and buffalo stomach lysozyme. Among several species, e.g., in human, chimpanzee, mouse, rat, cattle, buffalo, sheep and goat, orthologs shared high similarity. Identity of buffalo LYZL proteins was 96–100% with orthologs of ruminants, 75–88% with primate orthologs and 73–80% with rodent orthologs. Buffalo LYZL proteins showed highest sequence identity with cattle orthologs. SLLP1 and LYZL4 showed 100% identity, while LYZL5 showed lowest identity at 97% with the respective cattle proteins. LYZL2 and LYZL6 showed 99% identity with cattle orthologs. In most of the cases, buffalo LYZL2 showed highest identity, while LYZL5 showed lowest identity with their orthologs in primates and rodents.

**Fig 1:**
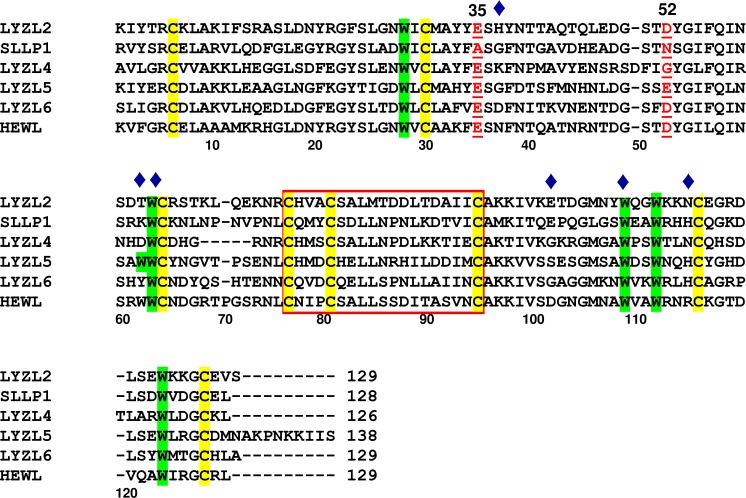
Multiple sequence alignment of deduced amino acid sequences of buffalo matured LYZL2, SLLP1, LYZL4, LYZL5, LYZL6 and HEWL. The sequence shown within the red color box indicates specific signature of lysozyme family. The conserved cysteine and tryptophan residues are highlighted with yellow and green color bars, respectively. The catalytic residues corresponding to positions 35 and 52 of c-type lysozyme are shown in red color and underlined. The residues marked with diamond (♦) in blue color represent substrate binding sites in c-type lysozymes.

Multiple sequence alignment of mature LYZL proteins showed that the eight cysteine residues forming four disulfide bonds in c-type lysozyme were conserved in all the buffalo LYZL proteins (Fig 1). LYZL5 contained an additional tail of 10 amino acids at the c-terminus (residues 129–138) as compared to other LYZL proteins. A deletion of five amino acids (residues 69–73) was observed in buffalo LYZL4. Similar deletion has also been observed in human, rat and mouse LYZL4 [18,30]. HEWL and most other non-testicular lysozymes contained six tryptophan residues that are conserved in only LYZL5, other LYZL proteins possessed only five conserved tryptophan residues. Some of the tryptophan residues are part of a hydrophobic box in c-type lysozymes. In HEWL, the box is made by Leu17, Tyr20, Tyr23, Trp28 and Trp111. All the buffalo LYZL proteins contained Leu17, Trp28 and Trp111. The residue numbering is as per HEWL, but due to addition and deletions, Trp111 has different residue number in LYZL proteins. Tyr20 was conserved in LYZL2 and SLLP1, but replaced with aromatic residue Phe in LYZL4, LYZL5 and LYZL6. Tyr23 was conserved in all LYZL proteins except in LYZL2, in which Phe23 was present. HEWL Trp62 is also conserved in turkey, buffalo, cattle, goat and sheep non-testicular lysozymes, while the non-testicular lysozymes in human, monkey, rat, mouse and pig contained tyrosine at position 62. Trp62 was replaced with Thr, Lys, Asp and Tyr in buffalo LYZL2, SLLP1, LYZL4 and LYZL6 proteins, respectively. PROSITE analysis revealed that all buffalo LYZLs contained the signature sequence pattern, (CxxxCxxxxxxxxxxxxxC), x- any amino acid) of lysozyme family. Same sequence pattern was also reported in case of human and mouse proteins [16,31].

### Catalytic residues in LYZL proteins

Catalytic residues glutamate 35 (Glu35) and aspartate 52 (Asp52) in lysozymes were substituted in some of the buffalo LYZL proteins (Fig 1). Glu35 and Asp52 are crucial for catalytic activity of lysozyme and are completely conserved in all non-testicular catalytically active lysozymes. Fig 1 shows that catalytic residues were substituted in SLLP1, LYZL4 and LYZL5 proteins, while both the residues were conserved in LYZL2 and LYZL6 proteins. Glu35 is conserved in LYZL2 in all the mammalian species considered in the study (S1 Table). Asp52 was present in all except platypus which possessed alanine (Ala52) at this position. Asparagine present at position 52 (Asn52) in SLLP1 was conserved in SLLP1 of all other species, while residue at position 35 was variable. Cattle, buffalo, goat, sheep, pig, marmoset and camel possessed alanine (Ala35), while most other species where sequences are available (human, gorilla, chimpanzee, gibbon, baboon, monkey, orangutan, ground squirrel, mouse, rat, rabbit, guinea pig, dog, cat, giant panda, mustela furo, african elephant, horse, little brown bat, black flying fox, chinese hamster, american pika, armadillo, mole rat and european shrew) possessed threonine (Thr35). A deviation in garnett’s greater bushbaby and platypus was seen, they contained Ser35 and Glu35, respectively in SLLP1. In LYZL4 Glu35 was conserved in all mammals except in platypus that has alanine, while second catalytic residue Asp showed Asp53→Gly replacement in buffalo and other mammalian species. On the other hand, LYZL4 in platypus and armadillo possessed serine (Ser53). In LYZL5 both 35 and 52 positions were occupied by Glu in all the species except little brown bat which possessed valine (Val35) and glutamine (Gln35). In case of LYZL6, both the catalytic residues (Glu35 and Asp52) were conserved in buffalo and other species, except in horse which has glutamine at position 35.

### Substrate binding pocket in LYZL proteins

The substrate binding pocket of lysozyme consists of residues including Asn37, Trp62, Trp63, Asp101, Trp108, and Arg114 in HEWL [2,16,31]. Three tryptophan namely Trp62, Trp63 and Trp108 play an important role in substrate binding [58,59]. Analysis of buffalo LYZL proteins showed that Trp63 and Trp108 were conserved in all the LYZL proteins as well; however, variations were observed in other substrate binding residues (Table 1). Trp63/108 were conserved in all the members of c-type lysozyme family as well, while variations have been observed at position 62 (Trp62) and other substrate binding residues [1,60]. Only LYZL5 possessed Trp62, which was replaced with aromatic Tyr in LYZL6, polar Thr in LYZL2, ionic Lys in SLLP1 and Asp in LYZL4. Polar Asn37 in HEWL was replaced with ionic Asp in LYZL6, polar His in LYZL2, and Gly in SLLP1 and LYZL5. Asp101 was replaced with Glu in LYZL2 and SLLP1, Ser in LYZL5, Gly in LYZL4 and LYZL6. Ionic Arg114 was replaced either with polar Asn in LYZL2 and LYZL4 or His in SLLP1, LYZL5 and LYZL6.

**Table 1:** Substrate binding residues in c-type lysozyme family.

Residue Number (according to HEWL seq)	Non-Testicular c-type Lysozymes	Testicular Lysozyme-Like Proteins from several species						
		LYZL2	SLLP1	SLLP1 (Reptiles)	LYZL4	LYZL5	LYZL5 (Frog)	LYZL6
37	N*/D/S/G/R (Cattle-intestine)	G*/H/R	G	G (Alligator, Anole)/H (Python, Garter snake)/R (Turtle)	K*/H (Dog)	G	R	K*/D/E (Pig)/H/N/R (Dog)/Y
62	W*/Y	T*/A/I (Platypus)/V (Orangutan)	K*/R/L (Platypus)	L*/I (Python)/Q (Turtle)	D*/E/A (Pika)/G (Bushbaby, Rabbit)/K (Guinea pig)/L (Squirrel)/V (Horse, Gibbon)	W	W	Y*/F (Pika, Rabbit)/V (Platypus)/L (European shrew)
63	W	W	W	W	W*/R (Molerat)	W*/C (Mole rat)	W	W
101	D*/G (Turkey)	E*/D (Pika, Rabbit)	E*/D/G/Q/R (Bushbaby)/V (Platypus)	G*/Q (Turtle, Alligator)	G*/D (Cat) /R (Flying fox)/E (Horse, Platypus)/T (Bushbaby)	S*/A (Squirrel)/L (European shrew, Hamster)/T (Rabbit)/Q (Platypus)	D	G*/A (Guinea pig)/E (Platypus)/R/S (Horse)
108	W	W	W*/R (Platypus)	W	W*/R (Dog)	W	W	W
114	H*/K/R	H*/N/R (Molerat)/Y (Elephant)	H*/N (Platypus)/	N*/H (Turtle, Alligator)	N*/F (Bushbaby)/H (Mole rat, Guinea pig)/Y	H*/N (Platypus)	Y	H*/N (Camel)/Y (Marmoset)

* The most occurring residues in species at respective positions, while those occurring in one or two species only are shown with species name in parenthesis.

Analysis of the substrate binding residues of LYZL proteins across vertebrate species showed large variation at several positions (Table 1), although few positions were much more intolerant to variation than others. Residues at sites 62, 101, 114 in LYZL4; 101 in SLLP1; 37, 62, and 101 in LYZL6 were more variable. Sites 63 and 108, both occupied by tryptophan, were least tolerant to variation. Position 62 represented by tryptophan in most cases or tyrosine in few of the non-testicular lysozymes was conserved in LYZL5 of most species. This position was represented by threonine or alanine in LYZL2, lysine or arginine in SLLP1, aspartate or glutamate in LYZL4, and tyrosine in LYZL6 of most of species. Trp63 was almost completely conserved in all orthologs of all the LYZL proteins except in case of LYZL4 and LYZL5 of molerat. Site 37 also showed lower tolerance to changes in SLLP1, LYZL4, and LYZL5, although this site showed higher variability in non-testicular lysozymes.

The replacement of residues involved in binding with residues of significantly different physicochemical properties suggested that either the chemical nature of these residues might not be very crucial for binding of substrates or they have evolved for different subset of receptors/ligands.

### LYZL proteins possess structural scaffold similar to c-type lysozymes

**Fig 2:**
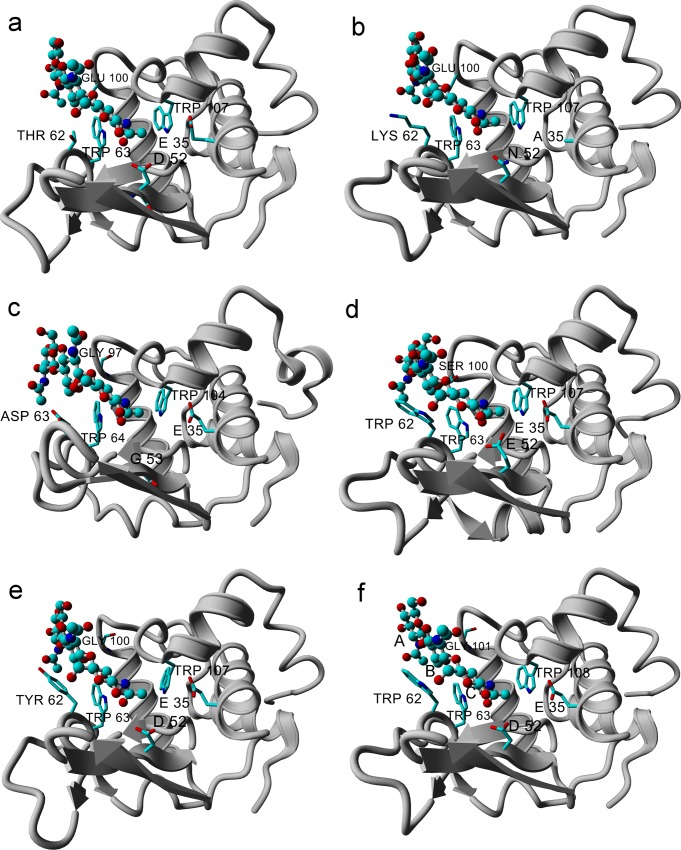
Structural models of lysozyme-like proteins complexed with (NAG)_3_ based on several template structures. PDB IDs of template are provided in material method part. For clarity only one model for each LYZL protein is shown. Panels a–LYZL2, b–SLLP1, c–LYZL4, d–LYZL5, e–LYZL6 and f– 1JEF (TEWL as one of the template). The protein part is shown in gray color and (NAG)_3_ molecule in ball and stick style has been shown in elemental colors. The substrate binding residues interacting with (NAG)_3_ are shown in three letter amino acid codes, while catalytic residues (residues at position 35 and 52 or 53) are labelled with single letter code. The NAG monomer binding subsites are represented by capital letters A, B and C in panel f.

Comparative models of various LYZL proteins complexed with (NAG)_3_ possessed structural topology similar to lysozyme (Fig 2). Mouse SLLP1 (mSLLP1) crystal structure (2GOI) solved at 2.30 Å has also been reported to contain glycan binding groove similar to lysozyme [61]. Buffalo SLLP1 shared 73% sequence identity with mSLLP1. Structural model of buffalo SLLP1 created with mSLLP1 template differed from SLLP1-(NAG)_3_ complex based on six other lysozyme structures with RMSDs of 0.9±0.05Å. The observed RMSD between models created by using different template structures with considerably different sequences suggested the reliability of homology modeling protocol.

The average RMSD between models of buffalo LYZL proteins and the respective template structures was 0.5–0.8 Å. The major difference in the structures of various LYZL proteins was observed in the loop spanning residues 65 to 75. The conformation of this loop was also variable in template structures. This loop in buffalo LYZL4 was shorter by 5 residues. On the other hand, an insertion of one residue (Asp) was observed at position 50 in the β-turn connecting two β-strands in LYZL4 (residues number 51–55). In all other LYZL proteins, the loop size in β-turn was conserved. Another significant feature was observed in LYZL5 protein, which contained 10 extra amino acid residues at the c-terminus in comparison to other LYZL proteins. No function has been assigned to the segment; however, the conservation of the segment in LYZL5 of all the species suggested its functional implication. The BLAST search of the segment sequence in NCBI database revealed 90% identity with ankyrin repeat domain-containing protein 27, also known as Vps9 and ankyrin repeat containing protein (Varp) [62]. Ankyrin repeat-containing proteins constitute a large family of proteins involved in cellular interaction, communication, adhesion, signaling, vesicle fusion and molecular trafficking [62,63].

### Analysis of substrate binding pocket of LYZL proteins

Fig 2 shows (NAG)_3_ complexed with LYZL proteins in the A-B-C mode in the substrate binding pocket of LYZL proteins, similar to those present in template structures [43]. The possibility of the binding of (NAG)_3_ in the B-C-D mode was explored if (NAG)_3_ shifts position from A-B-C to B-C-D; however, no shift was observed in the molecular dynamics trajectory of 5000 ps simulation in explicit solvent. The existence of the B-C-D binding mode could not be rejected given the short simulation time. Long simulation considerably changed the conformation of (NAG)_3_ and LYZL proteins. Therefore, this approach to explore existence of alternative binding mode was dispensed with and homology models were created based on template with (NAG)_3_ in the B-C-D binding mode and their binding energies were compared with those in the A-B-C mode.

In our calculations, the template complex showed an average binding energy of -5.7 kcal/mol for binding in the A-B-C subsites in the lysozyme-(NAG)_3_ complex in the template structures. The result represents template complex structures of wild or mutants of human lysozyme, HEWL [43,44,45] and TEWL [46]. In some cases, the mutations were present at substrate binding site [45]. Moreover, human lysozyme possessed Tyr62 and TEWL possessed Gly101 in place of Trp62 and Asp101 in HEWL. Both these residues are known to stabilize substrate binding [64]. Asp101 is considered important for stabilizing the substrate binding since the Asp101→Gly replacement has been shown to decrease binding energy in HEWL [2]. The presence of Gly101 in TEWL resulted in the lowest binding energy with (NAG)_3_ among all the template structures. This caused a marginally lower average binding energy of all templates together in comparison to HEWL. The human lysozyme has Tyr63 at position corresponding to Trp62 in HEWL, and the two molecules of human lysozyme 1BB5A and 1BB5B showed almost similar binding energy (-5.79 kcal/mol) with (NAG)_3_. In case of HEWL, the binding energies were similar for complex templates 1LZB (-6.02 kcal/mol) and 3A3Q (-5.87 kcal/mol) in the A-B-C binding mode. Our results suggested that the obtained values was consistent with the relative affinities of (NAG)_3_ for various lysozymes and analysis could be extended to LYZL proteins.

The experimental binding energy of (NAG)_3_ with HEWL has been reported to be -7.0 kcal/mol [65], which is higher than the calculated value of -5.95 kcal/mol (average of 1LZB and 3A3Q PDBs templates). Nonetheless, these empirical values should provide relative binding strength of (NAG)_3_ with various LYZL proteins. Table 2 lists the binding energies and contacting residues of LYZL proteins with (NAG)_3_. The binding energies of LYZL2, SLLP1 and LYZL4 were insignificantly smaller by ≤0.5 kcal/mol in comparison to HEWL in the A-B-C mode. In case of LYZL6, the binding energies with (NAG)_3_ in the A-B-C subsites was moderately lower (≥1.0 kcal/mol) than that of HEWL.

**Table 2:** Binding energy, contacting residues and hydrogen bonding residues of LYZL with (NAG)_3_ in their complex structures.

**LYZL-(NAG)_3_ complex**	**Binding Energy (kcal/mol) ±sd^a^**	**Contacting residues (ABC mode)^b^**	**Residues^c^ involved in hydrogen bonding with (NAG)_3_**
LYZL2-(NAG)_3_	5.50 ± 0.42	Asp52, Gln57, Phe56, Ile58, Asn59, Thr62, Trp63, Lys72, Arg74, Ile97, Glu100, Thr101, Asp102, Tyr106, Trp107, Gln108	Asn59, Thr62(8) Trp63, Glu100, Thr101(15), Asp102(8), Tyr106, Gln108(10)
SLLP1-(NAG)_3_	5.52 ± 0.45	Gln57, Ile58, Asn59, Arg61, Lys62, Trp63, Leu74, Ile97, Glu100, Pro101, Gln102, Ser106, Trp107	Asn59, Arg61(11), Trp63, Glu100, Gln102, Ser106
LYZL4-(NAG)_3_	5.43 ± 0.60	Phe57, Gln58, Ile59, Arg60, His62, Asp63, Trp64, Arg69, Arg71, Ile94, Gly97, Lys98, Arg99, Ala103, Trp104, Pro105	Arg60, Trp64, Arg71(14), Gly97(16), Arg99, Ala103
LYZL5-(NAG)_3_	5.32 ± 0.41	Glu52, Phe56, Gln57, Leu58, Asn59, Trp62, Trp63, Val97, Ser100, Glu101, Ser102, Ala106, Trp107, Asp108	Asn59, Trp62, Trp63, Glu101(25), Ala106, Asp108(15)
LYZL6-(NAG)_3_	4.83 ± 0.44	Asp52, Phe56, Gln57, Ile58, Asn59, Tyr62, Trp63, Asn74, Ile97, Gly100, Ala101, Gly102, Asn106, Trp107, Val108	Asn59, Trp63, Gly100(10), Gly102(8), Asn106
Template (1LZB)	6.02 ± 0.25	Asn46, Asp52, Gln57, Ile58, Asn59, Arg61, Trp62, Trp63, Arg73, Leu75, Ile98, Asp101, Gly102, Asn103, Ala107, Trp108	Asn59, Trp62, Trp63, Asp101, Asn103, Ala107
**(BCD mode)**^**b**^			
LYZL2-(NAG)_3_	6.84 ± 0.39	Glu35, Leu46, Asp52, Gln57, Ile58, Asn59, Thr62, Trp63, Arg74, Ile97, Glu100, Tyr106, Trp107, Gln108, Gly109	Glu35, Asp52, Gln57(10), Asn59, Thr62(11), Trp63, Glu100, Tyr106, Gln108
SLLP1-(NAG)_3_	7.34 ± 0.47	Glu46, Asn52, Gln57, Ile58, Asn59, Arg61, Lys62, Trp63, Leu74,0 Ile97, Glu100, Gln102, Ser106, Trp107, Glu108, Ala109	Glu46, Asn52(21), Gln57(17), Asn59, Arg61(13), Trp63, Glu100(18), Gln102, Ser106, Glu108
LYZL4-(NAG)_3_	7.62 ± 0.47	Glu35, Tyr44, Asn46, Arg48, Phe57, Gln58, Ile59, Arg60, His62, Asp63, Trp64, Arg71, Ile94, Arg99, Ala103, Trp104, Pro105, Ser106	Glu35, Asn46(25), Gln58, Arg60, Asp63(21), Trp64, Arg71(13), Arg99, Ala103
LYZL5-(NAG)_3_	6.93 ± 0.33	Glu35, Asn44, Asn46, Glu52, Phe56, Gln57, Leu58, Asn59, Trp62 Trp63, Val97, Ser100, Ser102, Ala106, Trp107, Asp108, Ser109	Glu35, Glu52, Asn59, Trp62(16), Trp63, Ser102(8), Ala106, Asp108
LYZL6-(NAG)_3_	6.69 ± 0.41	Glu35, Asn44, Asn46, Asp52, Phe56, Gln57, Ile58, Asn59, Tyr62, Trp63, Asn74, Ile97, Asn106, Trp107, Val108, Lys109	Glu35, Asn46(17), Asp52, Gln57(9), Asn59, Tyr62(6), Trp63, Asn106, Val108
Template (1LMP)	7.0 ± 0.34	Glu35, Asn44, Asn46, Asp52, Gln57, Ile58, Asn59, Tyr62, Trp63, Lys73, Val75, Val98, Asp101, Asn103, Ala107, Trp108, Val109, Ala110	Glu35, Asp52, Asn59, Tyr62, Trp63, Asp101, Asn103, Ala107, Val109

^**a**^ sd—standard deviation.

^**b**^ The listed residues were observed interacting with (NAG)_3_ in majority of the model structures. The residue numbering differ among the LYZL proteins due to insertions or deletions, see sequence alignment in Fig 1 for corresponding residues in various LYZL proteins. The data are based on analysis of 60 structures in each case of LYZL proteins in each of the binding mode.

^**c**^ Residues shown are those involved in making hydrogen bond with (NAG)_3_ in most of the model structures. The numbers in parenthesis indicate the number of models in which a particular residue was observed in only a few models making hydrogen bond with (NAG)_3_ out of total 60 models.

On the other hand, the binding energies of (NAG)_3_ was always higher in the B-C-D binding mode than in the A-B-C mode for each of the LYZL proteins. The binding in the B-C-D mode was favored by 1.3 kcal/mol to 2.2 kcal/mol in comparison to binding in the A-B-C mode (Table 2). These differences were significant and suggested that B-C-D binding mode should be more favorable for binding of (NAG)_3_ with LYZL proteins. Similar trend was also observed in case of template structures. Lysozyme templates showed a difference of >1 kcal/mol between the two modes. Several of the templates were created by deleting NAG residue at the subsites A or D from a lysozyme-(NAG)_4_ structure to create two binding modes and thus could have led to artefacts. To check it, we also calculated binding energy of HEWL-(NAG)_3_ created by deleting NAG at site D of template 1LZC and observed that binding energy was similar to that calculated for X-ray structure of HEW-(NAG)_3_ (1LZB). Similarly, binding energy of (NAG)_3_ in B-C-D subsites created from (NAG)_4_ complexed with RBTL (1LMQ) was similar to that obtained for X-ray structure of RBTL with (NAG)_3_ in B-C-D mode (1LMP). Consistency of these results suggested that the artificially created complexes of (NAG)_3_ with lysozymes did not introduce any artefact in the evaluated binding energies.

### Phylogenetic analysis of LYZL proteins

**Fig 3:**
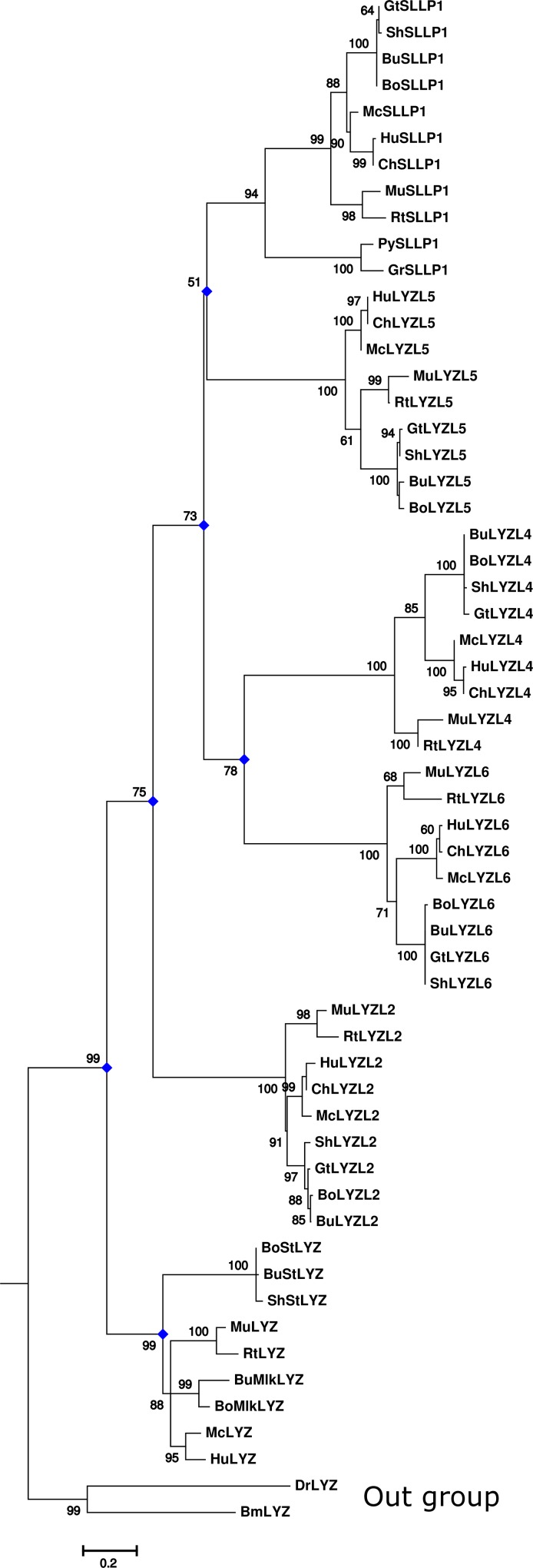
Phylogenetic map of lysozyme family. The phylogenetic tree was constructed by using ML method. The nodes with diamond (♦) in blue color represent duplication event. LYZ-lysozyme, LYZL-lysozyme-like proteins, BoSt-cattle stomach, BuSt-buffalo stomach, ShSt-sheep stomach, BoMlk-cattle milk, BuMlk-buffalo milk, Hu-human, Ch-chimpanzee, Mc-monkey, Rt-rat, Mu-mouse, Bo-cattle, Bu-buffalo, Sh-sheep, Gt-goat, Py-python, Gr-garter snake. Lysozymes from non-mammals such as drosophila (Dr) and *Bombyx mori* (Bm) were used as outgroups.

Gene tree was created with protein sequences by using ML method. Several substitution model for amino acid substitution were applied. Among them LG+G5+I evolutionary model provided the best likelihood values. Most of the important nodes showed reliable boostrap values of >70% except for the bifurcation of branch leading to SLLP1 and LYZL5 subtrees (Fig 3). The gene tree suggested the duplication of ancestral c-type lysozyme gene into testicular and non-testicular lysozymes, the later one bifurcated further in to stomach and milk lysozymes in ruminants. On the other branch, multiple duplications and the divergence of the testicular genes led to evolution of multiple forms of lysozyme-like genes. First duplication in the ancestral *Lyzl* gene led to *Lyzl2* gene on one branch and ancestral gene of others on another clade. Subsequent duplication of the later ancestral gene resulted into *Sllp1* and *Lyzl5* on one branch and *Lyzl4* and *Lyzl6* on another. The gene duplication event leading to evolution of *Lyzl4* and *Lyzl6* was reliable; however, the ancestral node of *Sllp1* and *Lyzl5* showed lower bootstrap value. Bayesian phylogenetic inference of the gene tree also suggested similar relationship of *Lyzl* genes (tree not shown) as shown by ML method. Gene tree clearly suggested that the duplication events leading to various LYZL paralog genes took place prior to speciation of mammalian species from reptiles and amphibians.

## Discussion

### Buffalo sperm possess all the known forms of LYZL proteins

All the known forms of LYZL proteins, namely LYZL2, LYZL3 or SLLP1, LYZL4, LYZL5 and LYZL6, were discovered in the buffalo sperm sub-proteome corresponding to 12–18 kDa molecular mass. Analyses of proteome of several species suggested that not all LYZL paralogs were present in sperm. However, this could also be because of inherent problems associated with efficiency of protein extraction, detection or identification associated with MS workflow. LYZL3 also named as SLLP1 has been detected in the sperm proteome of human [66–68], cattle [69,70] and horse [71]. LYZL4 has been detected in the sperm proteome of human [72,73], cattle [69,74] and boar [29]. The LYZL5/SPACA5 protein has been detected on human sperm [23,72]. Lysozyme, LYZL4, LYZL3/SPACA3 and LYZL5/SPACA5 were also identified in human sperm tail proteome [24].

Few of the sequenced genomes, especially those of mammals, suggested the presence of *Lyzl* genes. *Lyzl1* and *Lyzl2* are duplicated forms of the same gene and have been identified in only primates like human, chimpanzee, macaca, orangutan and common marmoset [15], while baboon possessed only a single gene. Both LYZL1 and LYZL2 have also been identified in human sperm proteome [21], which suggested that these genes could be functional copies. In northern treeshrew one copy of *Lyzl1* has been observed as functional, while the other one as a pseudogene [15]. *Spaca*5 has also undergone duplication in human, while most primates and other mammalian species contain only a single copy. In buffalo sperm proteome we could identify only one form of each of LYZL2 and LYZL5. In the extreme case, genome of grey short tailed opossum, a marsupial, has been reported to have undergone deletion of *Lyzl1*/*2*, *Lyzl3*/*Spaca3*, *Lyzl6* genes, whereas *Lyzl4* and *Lyzl5/Spaca5* genes could not be identified in their genome [15]. We cloned and sequenced the cDNA of all the five buffalo *Lyzl* genes, which should be functional since their translated products were also detected in the buffalo sperm proteome. We could also detect all these proteins in the goat sperm proteome as well (unpublished data). Cattle also possessed all *Lyzl* genes as revealed by the presence of cDNA sequences or genomic sequences in the NCBI nucleotide database. These data suggest that there should be a strong evolutionary pressure for retaining *Lyzl* genes in mammals and several other vertebrates at high level of sequence conservation.

### Bacteriolytic activity in LYZL proteins

Lysozyme has evolved in different tissues to perform a specific function. LYZL proteins evolved in testis in multiple forms. Mammary gland lysozymes are basic proteins having p*I* close to 9.0, whereas stomach lysozymes are acidic in nature. These proteins evolved and adapted to particular environment of the tissue. In case of testicular lysozymes, it is noteworthy that LYZL2 and LYZL4 are basic proteins, while others are acidic proteins. The presence of both acidic and basic LYZL proteins in the male reproductive system is not clear given the identical environment in which they have evolved. LYZL2 (basic) and LYZL6 (acidic) possessed intact catalytic residues and could be possessing bacteriolytic activity, which has been shown at least in case of human LYZL6 [19]. The other three LYZL proteins are likely to be inactive because of mutation of catalytic residues.

Human SLLP1 did not exhibit catalytic activity because of Glu35→Thr and Asp52→Asn mutations [16]. Buffalo SLLP1 has also undergone changes at both of these positions (Glu35→Ala and Asp52→Asn), which might render it catalytically inactive, that was also indicated by our preliminary finding on recombinant buffalo SLLP1 expressed in yeast (unpublished results). Lack of bacteriolytic activity has also been observed in case of rat and mouse LYZL4 proteins that could also be inferred from the fact that both Glu35 and Asp52 are essentially required for catalytic activity [18,30]. LYZL5 contained Glu at both 35 and 52 positions. Analysis of lysozymes (Asp52→Glu) mutant (Glu35/Glu52) showed <3% bacteriolytic activity as compared to its wild type [75,76], which implies that buffalo LYZL5 might also possess very low bacteriolytic activity, if any.

### Substrate binding pocket in LYZL proteins

Structural modeling of LYZL proteins with (NAG)_3_ showed that these proteins carry substrate binding cleft similar to that present in active c-type lysozymes. Computational analysis of (NAG)_3_ binding in the A-B-C subsites of various LYZL proteins showed binding energies of LYZL2 and SLLP1 almost similar to that of HEWL. LYZL4 and LYZL5 showed binding energy only moderately smaller by >0.5 kcal/mol and LYZL6 smaller by >1.0 kcal/mol in comparison to HEWL.

Asp101→Gly replacement in HEWL has been reported to change the conformation of the main-chain that forms a loop structure, which is part of the active site around subsite A. The loss of hydrogen bonding between (NAG)_3_ and Asp101 in Asp101→Gly HEWL mutant has been observed to reduce the stacking interaction of Trp62 with (NAG)_3_ [63]. Double mutants Trp62→His/Asp101→Gly [60] and Trp62→Tyr/Asp101→Gly [2] reduced the substrate binding affinity significantly. TEWL possesses Gly101 and the loop conformation has been observed similar to that seen in Asp101→Gly mutant of HEWL [46,64]. LYZL4 and LYZL6 have undergone Asp101→Gly replacement, while LYZL5 contains serine at corresponding position (Fig 2). These replacements are likely to affect the binding energy of (NAG)_3_ with LYZL proteins at subsite A in the A-B-C mode. Except LYZL5, all the LYZL proteins have undergone replacement at position 62 (Table 1), which could also affect their affinities for (NAG)_3_ in the A-B-C subsites. Trp62 mutant of HEWL showed enhanced rate of catalysis as well as dissociation constant [2,77–80]. Although Trp62 is important for cushioning and stabilizing substrate molecule, yet position 62 seems comparatively more tolerant to changes. We observed that position 62 is occupied by residues with dissimilar physicochemical properties in lysozymes of various species (Table 1). LYZL2 and SLLP1 possessing Glu100 showed higher affinity for (NAG)_3_, which suggested that the presence of Asp or Glu at this position is important to stabilize binding of (NAG)_3_ with LYZL proteins. Aspartate at this position could make up to two hydrogen bonds with (NAG)_3_ to stabilize the protein-carbohydrate complex. LYZL6 was observed to make least number of hydrogen bonds with (NAG)_3_ and also showed lowest binding energy (Table 2).

All the buffalo LYZL proteins possessed a conserved Trp at position corresponding to Trp63 of HEWL. This residue also makes extensive interaction with (NAG)_3_ and replacement of Trp62 and/or Asp101, as in case of LYZL proteins, is likely to change the interaction of Trp63 with (NAG)_3_ as well. Given that Trp63 is almost 100% conserved in all LYZL proteins across the species except in case of molerat (Table 1), the residues corresponding to HEWL at position 62 and 101 should be deciding factor for the stability of complex. Previous studies suggested that even a single or double mutation in substrate binding pocket of lysozyme could affect the binding affinity of lysozyme for their substrates to varying extent [2,43,60, 64,81,82], while still maintaining the overall structure almost identical to wild type [51,64].

Binding energies of (NAG)_3_ with LYZL proteins in the B-C-D mode were always higher than in A-B-C mode. Our results implied that binding of (NAG)_3_ with LYZL proteins in B-C-D mode could be energetically more favorable, which is consistent with HEWL-(NAG)_3_ showing higher binding energy in B-C-D mode [83]. These results implied that a dynamic equilibrium between population of A-B-C and B-C-D subsites occupied LYZL proteins might be existing as observed in the crystal structures of Trp62 and Asp101 mutants of HEWL [43]. However, the X-ray crystallographic studies showed wild type and some mutants of HEWL, TEWL and human lysozymes to contain (NAG)_3_ in the A-B-C subsites [43–46]. This could be because of constraints placed by crystallization conditions or crystal formation that A-B-C subsites are preferred, since NMR [84] and enzymatic studies [85] suggested the occupancy of D subsite with (NAG)_3_. HEWL Tyr62→His mutant showed enhanced catalysis of (NAG)_3_ [86], which implied that (NAG)_3_ might be binding deeper inside the pocket to enable breakdown of (NAG)_3_ between subsites D and E. Powder diffraction [87] and MD simulation studies [83] also suggested binding of (NAG)_3_ in B-C-D mode to be energetically more favorable. Powder diffraction showed occupancy of A-B-C and B-C-D subsites existing in a ratio of 35:65, implying that B-C-D mode might be favorable. Our calculations on buffalo LYZL proteins also suggested B-C-D subsites to be more favorable for binding of (NAG)_3_. Analysis of hydrogen bonding network indicated that in B-C-D mode two or three more residues were involved in hydrogen bonding than in A-B-C binding mode in LYZL-(NAG)_3_ (Table 2). Hydrogen bonding is an important contributor to protein stabilization [88] as well as protein-carbohydrate stability [43] and excess of hydrogen bonding could cooperatively stabilize LYZL-(NAG)_3_ complex in the B-C-D binding mode.

Trp108 in HEWL and corresponding Trp in LYZL proteins are also completely conserved and known to make contact with substrate molecule (Table 1) as well as stabilize Glu35 through van der Waals interactions. Trp108 also contribute significantly to the overall stability of the molecule and Trp108→Tyr and Trp108→Gln mutants are reported to decrease not only conformational stability of the molecules but also catalytic activity and binding energy with the substrate molecules [76]. In human and HEWL, mutation of Arg114, also one of the residues involved in substrate binding, to His, Gln, Glu and Ala reduced the binding affinity [89–92]. In case of buffalo LYZL proteins, Arg114 is mutated to His in SLLP1, LYZL5 and LYZL6 and to Asn in LYZL2 and LYZL4. (NAG)_3_ is a small molecule and it does not make contact with residue corresponding to position Arg114 and hence might not be of much consequence in the calculated binding energy with LYZL proteins. Similarly, residue number 37 (Asn 37 in HEWL) also did not show interaction with (NAG)_3_ in both A-B-C and B-C-D binding modes and should not contribute in binding affinity toward LYZL proteins. This is consistent with the analysis of mutants Asn37→Gly and Asn37→Ser showing no significant change in binding constant with (NAG)_3_ [2,93]. Nevertheless, these residues might be involved in interaction with larger receptors like molecules on oocytes. It is interesting to note that despite the large sequence divergence of LYZL proteins among themselves and from non-testicular lysozymes, many contacting residues in the binding pocket are conserved (Table 2). This could be necessitated for the conservation of functional binding efficacy of the LYZL proteins in sperm-oocyte or other cellular interactions.

Crystal structure analysis of mSLLP1 suggested the presence of glycan binding groove similar to that in lysozyme [61]; however, only partially conserved in the central cavity. The surface charges present in the groove also differed between the two structures. *In silico* docking suggested unfavorable interaction of mSLLP1 with (NAG)_4_; however, binding with NAG and (NAG)_2_ was preserved. In case of buffalo LYZL proteins, structural models showed binding with (NAG)_3_ in two binding modes almost similar to those observed for non-testicular lysozymes. The mSLLP1 and human SLLP1 did not show significant binding with chitin or different glycans in a glycoarray [61]. The binding affinity of mSLLP1 and lysozyme also differed for glycan moieties. A new oocyte-specific receptor SAS1B has also been characterized that binds with sperm SLLP1 and helps in sperm-oocyte interaction [94]. These results indicated that LYZL proteins might have evolved for interaction with other receptors as well.

### Evolution of LYZL proteins and their functional significance

Sequence analysis showed that LYZL proteins have undergone large divergence from each other and non-testicular lysozymes. Testicular *Lyzl* genes might have evolved from the ancestral genes through several gene duplications and accumulation of mutations to perform specific functions in the male reproductive system. The presence of most forms of LYZL proteins with high identity with orthologs but moderate with paralogs (38–50%) from diverse species suggested several events of duplication before the divergence of these species (Fig 3). Divergence might have resulted into loss or gain of function or both together as has been the case of evolution of α-lactalbumin from lysozymes [13,95]. Non-testicular lysozyme provide the innate immunity against the pathogens in various tissues or as a digestive enzyme in the stomach of ruminants, leaf-eating monkey and birds [5,7,8]. Testicular lysozyme family members might have evolved to facilitate reproduction [16,30,31] as well as protection against bacterial pathogens due to the presence of catalytic activity, e.g., LYZL6 has been observed biologically active [19].

The evolutionary pressure responsible for the existence of multiple forms of LYZL proteins and their large scale diversity in testis is not clear. Preincubation of mouse sperm with antibodies against SLLP1 [31] and LYZL4 proteins [30] reduced fertilization by 61% and 80%, respectively. The SLLP1 receptor SAS1B gene knockout female mice showed a decrease in fertilization only by 34% [94], which suggested that other factors are also important. It is also not clear if each member of LYZL proteins has evolved to carry out a specific function that might be supplemented by other members or not. The presence of similar set of LYZL proteins with high sequence similarity of orthologs among distant mammals suggests that each member might be playing important role in the reproduction or the protection of organisms. The localization of several of LYZL proteins on the surface of sperm suggest that these proteins might be involved in events mediated through surface contact. SLLP1 has been reported to interact with zona pellucida 3 protein [96] and implicated in sperm-oocyte binding [16,31].

Similarly, LYZL4 has also been implicated in fertility [30] and LYZL5 proposed as a biomarker of fertility [28]. Additionally, 10 extra c-terminal residues observed in LYZL5 shared 90% similarity with ankyrin repeat domain-containing protein 27. Ankyrin are known to be involved in many cellular events involving vesicle trafficking, vesicle fusion, molecular recognition and various signaling pathways [63]. The role of extra residues in LYZL5 is not discernible given a much smaller size of only 10 residues in comparison to 33 residues in an ankyrin repeat domain [62,63]. Nevertheless, the presence of extra c-terminal sequence showing high similarity with mammalian Varp protein in almost all LYZL5 proteins of mammalian species could be suggestive of its important role in sperm function.

The likelihood of the involvement of LYZL proteins in protection against pathogens seems weak, since had it been the case LYZL proteins would have been more efficient in providing protection by being freely soluble in the seminal fluids rather than immobilized on the sperm surface. Moreover, functionally active lysozyme has also been detected in the seminal fluid to inhibit pathogens in testis [97] and thereby rendering LYZL proteins redundant in tissue protection. Nevertheless, the role of catalytically active LYZL proteins in innate immunity along with other function cannot be denied.

In SLLP1 of alligator and turtle, the catalytic residues are Glu35 and Asp52, which are similar to that present in catalytically active lysozymes. The SLLP1 of reptiles like python, cobra, garter snake and green anole are intermediate with only Asp52 replaced by Ser52, while Glu35 remaining conserved. Nevertheless, the SLLP1 in these species could be inactive as suggested by mutational analysis in active lysozymes [49]. The tissue-specific expression of SLLP1 in reptiles, and that if SLLP1 is a sperm-specific protein in reptiles is not known. The presence of only one *Lyzl* gene variant in reptiles or amphibians could be due to incomplete genome annotation or subsequent loss of some variants as has also been observed in some mammals [15]. It seems that in mammals SLLP1 and few other LYZL proteins diverged further and lost both the catalytic residues for specific function in reproduction. Sequence analysis suggests that SLLP1 in most of mammals is likely to be inactive because of mutations of both the residues. This has been shown in human [16] and buffalo (unpublished results). Unlike in mammals, there seems no study to implicate SLLP1 in the reproduction of reptiles. However, given the conserved catalytic residues in reptiles, it is not clear if the lack of catalytic activity in SLLP1 is not a pre-requisite for a specific role in reproduction or reptile SLLP1 underwent other adaptations to circumvent the requirement. The presence of conserved catalytic residues in reptiles also suggested that the loss of activity in SLLP1 should be a post speciation event. Similarly, the presence of some of the *Lyzl* genes in highly diverged extant vertebrates suggested that these paralogs should have diverged well before speciation of mammals from other vertebrates. However, it is not clear why LYZL2, which existed before the appearance of SLLP1 and LYZL5, have not been found in the genome of reptiles or frogs.

LYZL2 in almost all the mammalian species has conserved Glu35 and Asp52, except in platypus which has Ala52. These amino acid residues are encoded by GAA (Glu) and GAU (Asp) codons in milk and stomach lysozyme genes in buffalo and cattle. However, in buffalo LYZL2 and LYZL6, the corresponding codons are GAG and GAC identical to that observed in HEWL. The codon usage for the catalytic residues was also found conserved in human, mouse, cattle, and sheep LYZL2. In case of LYZL6, GAA (Glu35) is present in case of human and mouse instead of GAG as observed in buffalo, cattle and sheep, while GAC (Asp52) is conserved in all these species. Glu35 conserved in LYZL4 and LYZL5 is also encoded by GAG. It is noteworthy that all the conserved codons encoding the catalytically active residues (Glu35/Asp52) in buffalo LYZL proteins are identical to those observed in HEWL and not to the milk and stomach paralogs of buffalo. It suggests that the milk and stomach lysozymes have undergone evolutionary adaptation after duplication, while sperm lysozyme-like protein genes maintained the ancient codons identical to those present in distant vertebrates. In other cases, where the catalytic residues have been replaced, it was observed that in most of cases the change in amino acids was the result of a single or at the maximum two mutational events in the codons. Sequence variation also takes place to compensate a previous mutation to preserve the function [98]; nevertheless, large sequence variation is likely to cause some structural changes to enable specific function in the particular organ. In case of testis, the lysozyme type scaffold could have been selected by nature for molecular recognition of surface markers like glycan moieties on oocytes [31,96].

## Supporting Information

S1 Table Species wise list of lysozyme-like proteins taken in the present study. (PDF)
