# Supplementary material for: Structural, Functional and Phylogenetic Analysis of Sperm Lysozyme-Like Proteins
Source: PLoS One. 2016 Nov 10;11(11):e0166321. doi: 10.1371/journal.pone.0166321 (PMC5104373; doi:10.1371/journal.pone.0166321)
Supplement: S1 Table — (PDF) [file pone.0166321.s001.pdf]

S1 Table. Species wise list of lysozyme-like proteins taken in the present study.

	Species	LYZL1/ LYZL2	SLLP1	LYZL4	LYZL5	LYZL6
**Mammals**						
Primates	Human	●	●	●	●	●
	Chimpanzee	●	●	●	●	●
	Orangutan	●	●	ɷ	●	●
	Gorilla	●	●	●	●	●
	Baboon	●	●	●	●	●
	Monkey	●	●	●	●	●
	Giboon	●	●	●	●	●
	Marmoset	●	●	●	●	●
	Bushbaby	●	●	●	●	●
Monotremata	Platypus	●	●	●	ɷ	●
Rodentia	Mouse	●	●	●	●	●
	Rat	●	●	●	●	●
	Guinea pig	●	●	●	●	●
	Squirrel	●	ɷ	●	●	●
	Molerat	●	●	ɷ	●	●
	Chinese hamster	●	●	●	●	●
Artiodactyla	Buffalo	●	●	●	●	●
	Cattle	●	●	●	●	●
	Goat	●	●	●	●	●
	Sheep	●	●	●	●	●
	Camel	●	●	●	●	●
	Pig	●	●	●	●	●
Carnivora	Cat	●	●	●	●	
	Dog	●	●	●	●	●
	Mustela		●	●	●	●
	Giant panda	●	●	●	●	ɷ
Logomorpha	American pika	●	●	●		●
	Rabbit	●	●	●	●	●
Proboscidea	Elephant		●	●	●	●
Cingulata	Armadillo	●	●	●	●	
Chiroptera	Little brown bat		●	●	●	●
	Flying fox	●	●	●	●	●
Eulipotyphla	European shrew		●	●	●	●
Perrisodactyla	Horse	●	●	●	ɷ	●
**Reptiles**						
Squamata	Python		●			

	King cobra		●			
	Garter snake		●			
	Green anole		●			
Crocodilia	Alligator		●			
Testudines	Green sea turtle		●			
**Amphibia**						
Anura	Frog				●	

ω Incomplete sequence
